# Axial Compressive Behaviour of Square Through-Beam Joints between CFST Columns and RC Beams with Multi-Layers of Steel Meshes

**DOI:** 10.3390/ma13112482

**Published:** 2020-05-29

**Authors:** Weining Duan, Jian Cai, Xu-Lin Tang, Qing-Jun Chen, Chun Yang, An He

**Affiliations:** 1 School of Civil Engineering and Transportation, South China University of Technology, Guangzhou 510640, China; duan.wn@mail.scut.edu.cn (W.D.); cvjcai@scut.edu.cn (J.C.); qjchen@scut.edu.cn (Q.-J.C.); 2 State Key Laboratory of Subtropical Building Science, South China University of Technology, Guangzhou 510640, China; 3 Guangzhou Jishi Construction Group Co., Ltd., Guangzhou 510115, China; xulintang@foxmail.com; 4 School of Civil and Environmental Engineering, Nanyang Technological University, 50 Nanyang Avenue, Singapore 639798, Singapore; he.an@ntu.edu.sg

**Keywords:** through-beam joint, concrete filled steel tube (CFST) columns, reinforced concrete (RC), axial compressive behaviour, steel mesh, local compression, confined concrete, height factor

## Abstract

The axial compressive behaviour of an innovative type of square concrete filled steel tube (CFST) column to reinforced concrete (RC) beam joint was experimentally investigated in this paper. The innovative joint was designed such that (i) the steel tubes of the CFST columns were completely interrupted in the joint region, (ii) the longitudinal reinforcements from the RC beams could easily pass through the joint area and (iii) a reinforcement cage, including a series of reinforcement meshes and radial stirrups, was arranged in the joint area to strengthen the mechanical performance of the joint. A two-stage experimental study was conducted to investigate the behaviour of the innovative joint under axial compression loads, where the first stage of the tests included three full-scale innovative joint specimens subjected to axial compression to assess the feasibility of the joint detailing and propose measures to further improve its axial compressive behaviour, and the second stage of the tests involved 14 innovative joint specimens with the improved detailing to study the effect of the geometric size of the joint, concrete strength and volume ratio of the steel meshes on the bearing strengths of the joints. It was generally found from the experiments that (i) the innovative joint is capable of achieving the design criterion of the ‘strong joint-weak member’ with appropriate designs, and (ii) by decreasing the height factor and increasing the volume ratio of the steel meshes, the axial compressive strengths of the joints significantly increased, while the increase of the length factor is advantageous but limited to the resistances of the joint specimens. Because of the lack of existing design methods for the innovative joints, new design expressions were proposed to calculate the axial compression resistances of the innovative joints subjected to bearing loads, with the local compression effect, the confinement effect provided by the multi-layers of steel meshes and the height effect of concrete considered. It was found that the proposed design methods were capable of providing accurate and safe resistance predictions for the innovative joints.

## 1. Introduction

The concrete-filled steel tube (CFST) structure is a high-efficiency solution for high-rise buildings and bridges due to its high bearing capacity, excellent ductility and great energy dissipation capacity [1]. In CFST structures, the joints connecting the CFST columns and reinforced concrete (RC) beams play an important role in achieving the structural integrity of the CFST frame [2,3], and thus researches related to the detailing of the joints have become a hot spot during the past few years. One of the typical joints connecting the CFST columns and RC beams is the through-column joint, in which steel cleats are attached to the outer surface of the steel tube of the CFST column at the joint area and the longitudinal reinforcements in the RC beams were welded on these steel cleats. The seismic behaviour and the failure modes of the through-column joints have been widely investigated [4,5,6,7], indicating that the through-column joints possess excellent seismic behaviour but require a significant amount of site welding. 

**Figure 1:**
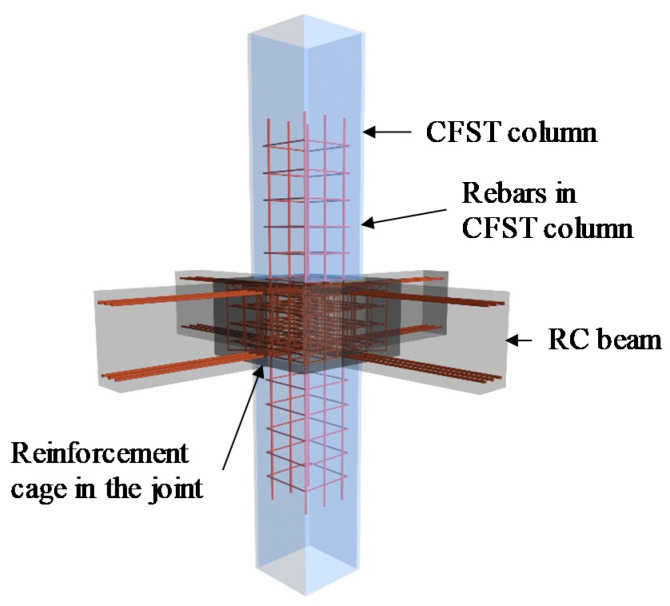
Square through-beam joint system.

Recently, through-beam joints connecting the CFST columns and RC beams have attracted the interest of researchers because of their simple detailing in the joint area, with a brief review of the recently developed through-beam joints summarised herein. Nie et al. [8] and Bai et al. [9] developed a representative through-beam joint connecting the concrete encased CFST columns and RC beams, where the steel tubes of the CFST columns were completely interrupted in the joint area, and the steel-reinforcement bars in the RC beams were continuous in the floor. Zhang et al. [10] developed a through-beam joint for concrete-filled double-skin steel tubes structural members, where the outer tube was interrupted in the joint area and an octagonal RC ring beam was arranged outside the joint to connect the reinforcements from the RC beams. Chen et al. [11,12] reported a new type of through-beam connection for CFST columns and RC beams, where the steel tube of the CFST column is completely or partially interrupted, and a ring beam was used to strengthen the load-carrying capacity of the joint. Zhou et al. [13,14] proposed a tubed-reinforced-concrete column to RC beam joint, where the steel tubes are completely interrupted in the joint area, with different manners to strengthen the joint, including the strengthening stirrup, horizontal hunches, and internal diaphragm. The seismic behaviour of the aforementioned through-beam joints was examined through cyclic loading tests. It was generally found that the through-beam joints are capable of achieving the design criterion of ‘strong joint-weak member’ with excellent seismic performance. Provided that the steel tubes of the CFST columns were generally interrupted at the joint area, axial compressive tests on the through-beam joints were also conducted by Nie et al. [8], Bai et al. [9], Chen et al. [11] and Zhou et al. [13], to investigate the axial compressive strengths of the joints; it was found that the strengths of the joints were higher than those of the corresponding CFST columns, and thus the design criterion of the ‘strong joint-weak member’ was deemed to be achieved. Recently, an innovative joint connecting the square CFST columns and RC beams has been developed by the authors [15]. The configuration and detailing of the innovative joint are displayed in Figure 1, where the outer steel tube was completely interrupted in the joint zone, and the longitudinal reinforcements from the RC beams could easily pass through the joint area. A reinforcement cage, including a series of reinforcement meshes and radial stirrups, was arranged in the joint area to strengthen the mechanical performance of the joint because of the interruption of the steel tube. The benefits of the proposed configuration of the innovative joints over the existing through-column and through-column beam joints include (i) the reinforcements from both structural columns and beams could easily pass through the joint area, ensuring both robustness and integrity of the joints, and (ii) the reinforcement cage in the joint can be precast and on-site welding is not required, leading to a simplified construction process. Cyclic loading tests on the innovative joints have been conducted by the authors [15] to study their seismic behaviour, while the axial compression behaviour of the innovative joints subjected to bearing loads remained unexplored and will be studied in the present paper. 

Because of the lack of existing experimental investigation on the compressive behaviour of innovative joints, a two-stage experimental study was first conducted to investigate the compressive behaviour of the innovative joint subjected to bearing loads, where the first stage of the tests included three full-scale innovative joint specimens to assess the feasibility of the joint detailing and propose measures to further improve the axial compressive behaviour of the joint, and the second stage of the tests involved 14 innovative joint specimens with the improved detailing to further study the effects of the geometric size of the joint, concrete strength and volume ratio of the steel meshes on the bearing strengths of the joints. Because of the lack of existing design methods for innovative joint, new design expressions for the bearing strengths of the innovative joints were developed and validated by the experimental results.

## 2. Tests of Joint with CFST Columns (Series I Tests)

### 2.1. General

**Figure 2:**
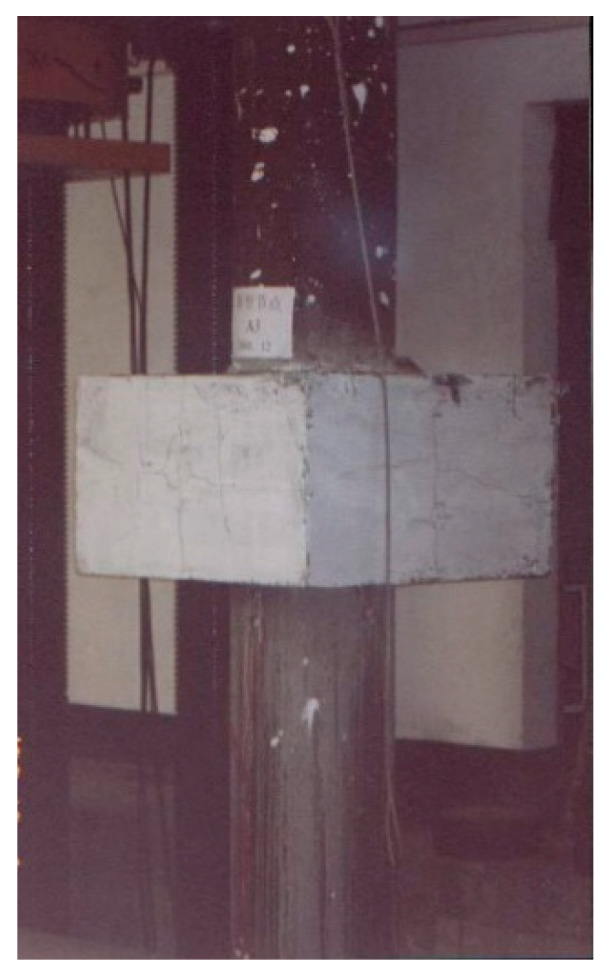
Overview of the specimens.

**Figure 3:**
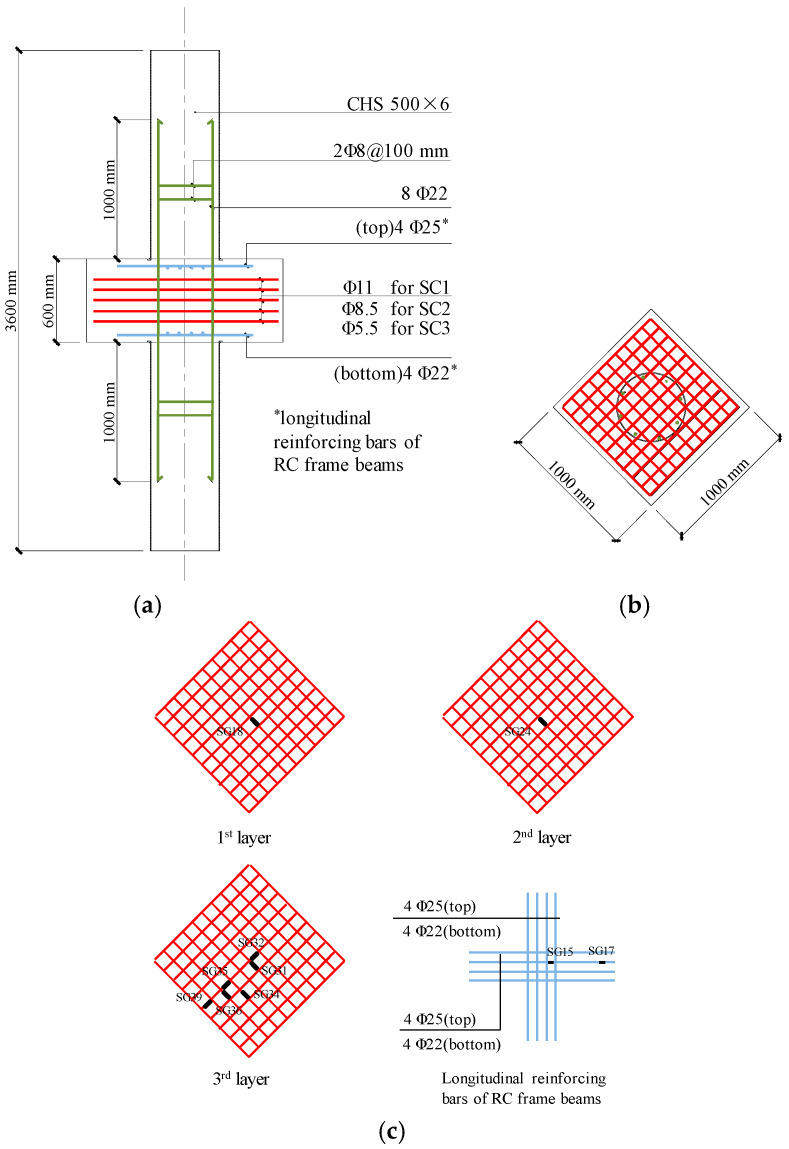
Details of the specimens in Series I (**a**) Elevation view; (**b**) Top view; (**c**) Steel meshes and beam rebars in the joints (SG: strain gauge).

The Series I axial compression tests included three geometrically identical full-scale specimens, namely SC1, SC2, and SC3, and were designed with various rebar diameters of the steel meshes in the joints. Each specimen included the upper and lower CFST columns and the joint, as shown in Figure 2, while the slab and the RC beams were not fabricated because of the size limitation of the testing machine. Note that the longitudinal reinforcements of the RC frame beams were still arranged in the joint. Figure 3 and Table 1 report the geometric dimensions and reinforcements of each part of the specimens. Specifically, the longitudinal reinforcements of the column were inserted through the joint area with the anchorage lengths of 1000 mm in the CFST columns, as determined based on the Chinese building code [16]. To avoid the compressive stress concentrating at the end of the steel tube, a steel ring whose width and thickness were 18 mm and 6 mm, respectively, was welded to each end of the steel tubes. Five layers of steel meshes with the adjacent distance of 75 mm were arranged in the joint area. The diameters of the rebars in the steel meshes were 11 mm, 8.5 mm and 5.5 mm for specimens SC1, SC2 and SC3, respectively, as shown in Figure 3.

During the fabrication of the specimens, four concrete cube specimens were respectively reserved when casting the concrete of the lower CFST column, joint area and upper CFST column. Compression tests were conducted on the concrete cubes to derive their cubic strengths at the day of the column tests, with the average cubic compressive strength of each part of the specimen reported in Table 1. Tensile tests were conducted to derive the material properties of the reinforcements and the steel tube, with the key results from the tensile tests reported in Table 2. The Young’s modulus of the steels may be taken as 200,000 MPa, according to the Chinese building code [16].

### 2.2. Experimental Test Setup

**Figure 4:**
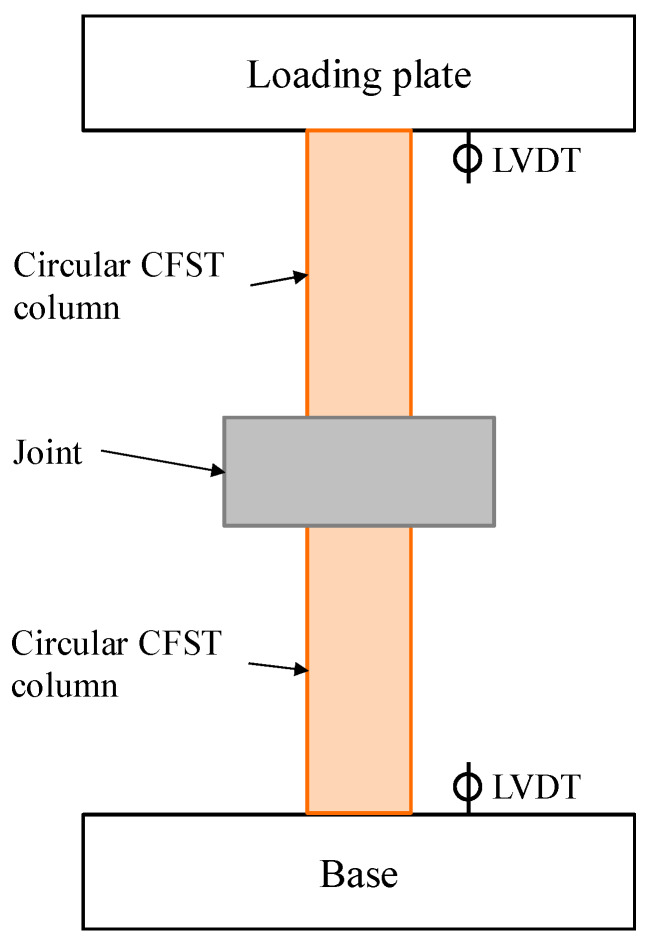
Schematic diagram of the test setup.

The Changchun CSS-254 15 MN universal testing machine was employed to apply axial compression force to the specimens, with the test setup shown in Figure 4. During the tests, four LVDTs were arranged in the joint area and the CFST columns to measured axial shortening of the specimen (see Figure 4), and a series of strain gauges were attached to the steel meshes in the joint to measure their deformations during loading (See Figure 3). Both load and displacement control modes were employed to drive the testing machine. Specifically, the initial loading rate was set to be equal to 5 kN/s until the material yielding was observed, according to the readings from the attached strain gauges, after which a displacement rate of 2 mm/min was used to complete the post-yield loading stage. The tests were terminated when the resistance of the specimen had deteriorated below 85% of its maximum strength.

### 2.3. Experimental Results

#### 2.3.1. Failure Modes

**Figure 5:**
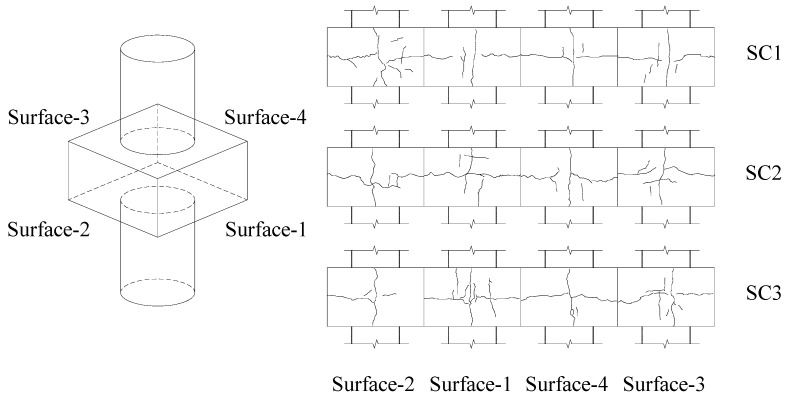
Cracks on the side surfaces of the specimens.

All the specimens exhibited similar overall behaviour under axial compression during the tests, as summarised below. The first vertical crack was found on the side surface of the joint when the upper load reached 44~57% of the peak loads. With the load increased, the horizontal cracks occurred and gradually crossed the first vertical crack. The dilation and local buckling of the steel tubes were observed when the specimens reached their peak loads, while the concrete in the joint area remained intact without series spalling, which indicates that the specimens were failed by the local buckling of outer steel tubes of the CFST columns and thus satisfied the design criterion of the ‘strong joint-weak member’. The crack patterns on each side surface of the joint at failure are shown in Figure 5.

Taken specimen SC2 as an example to illustrate the experimental observation in detail, when the load reached about 45% of *N*_u_ (*N*_u_ is the peak load of the specimen, for SC2 *N*_u_ = 12,004 kN), the first crack was found at the middle of the side surface of the joint in the vertical direction. As the load increased to about 50% of *N*_u_, the vertical crack developed with a maximum width of about 0.1 mm, and a short horizontal crack occurred on the side surface of the concrete at the same time. At about 70% of *N*_u_, the maximum widths of the vertical and horizontal cracks on the side surface of the joint reached 0.25 mm and 0.15 mm, respectively. When the specimen reached its maximum load *N*_u_, dilation and local buckling of the steel tubes of the CFST columns were observed. At the post-peak loading stage, the steel tubes of the CFST columns continued to dilate with the load capacity of the specimen gradually dropped to about 85% *N*_u_.

#### 2.3.2. Load-Deformation and Load-Strain Curves

**Figure 6:**
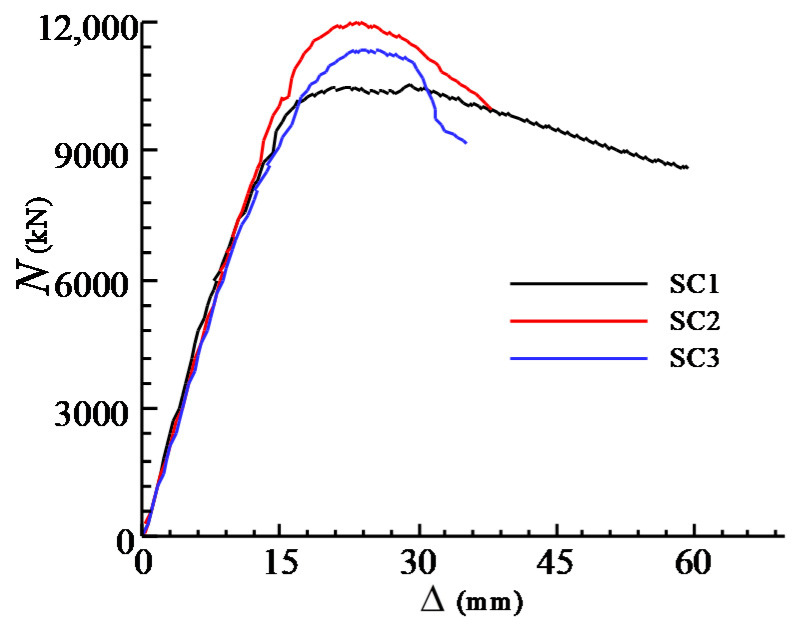
Axial load and displacement relationship.

The axial load-deformation curves of the three specimens are shown in Figure 6, where the vertical axis is the force applied at two ends of the columns, and the horizontal axis is the displacement of the whole specimen consisted of the upper and lower CFST columns and the joint zone. As shown in Figure 6, the initial elastic stiffnesses and the shapes of the curves for the three specimens are quite close to each other at the elastic stages. After reaching the peak loads of the specimens, the load-deformation curves descend gradually and show ductile post-peak behaviour. The loads corresponding to the first cracks *N*_cr_ and the peak loads *N*_u_ for all specimens are listed in Table 1. It can be seen that the value of *N*_cr_ for SC1 is 1.13 and 1.18 times than those of SC2 and SC3 respectively, which indicates that the crack resistance of the joint is enhanced by improving the volume ratio of steel meshes in the joint (*ρ*_v_). However, the ultimate axial compressive strength of SC1 is smaller than those of SC2 and SC3. It may be attributed to the discreteness of concrete material in the CFST columns, as all specimens eventually failed in the CFST columns.

**Figure 7:**
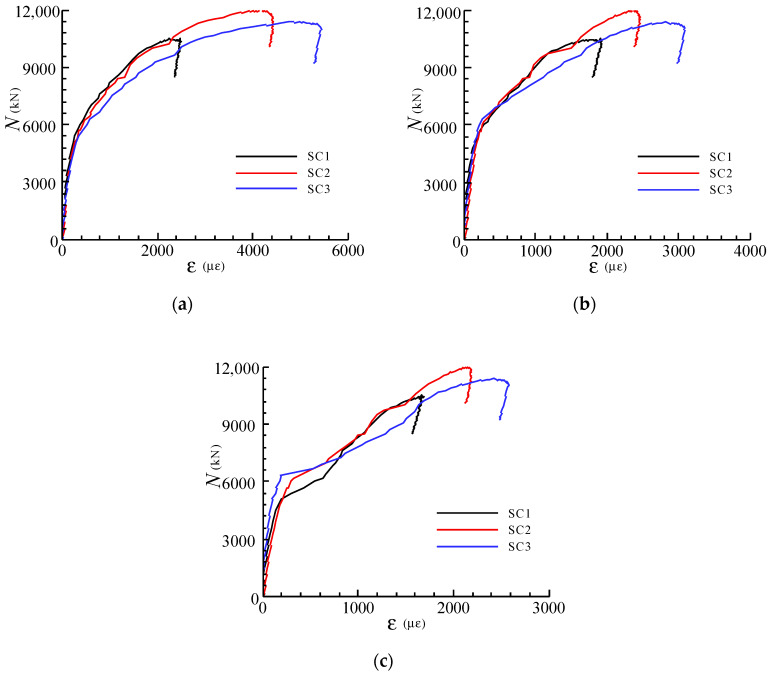
Load (*N*)-strain (*ε*) curves for steel meshes (**a**) SG24; (**b**) SG34; (**c**) SG36.

The load-strain relationships of the steel meshes in all specimens are similar, as Figure 7 shows. It can be found that the strains of the steel meshes grew slowly in the early loading phase. As the upper load increased to about 50% of *N*_u_ when the first crack appeared, the strains developed dramatically and subsequently reached the yield strains at the peak loads. It was also found that as the volume ratio of the steel meshes in the joint decreases, the strain values corresponding to the peak loads increase, which exhibits more adverse elongation deformation of the steel meshes.

**Figure 8:**
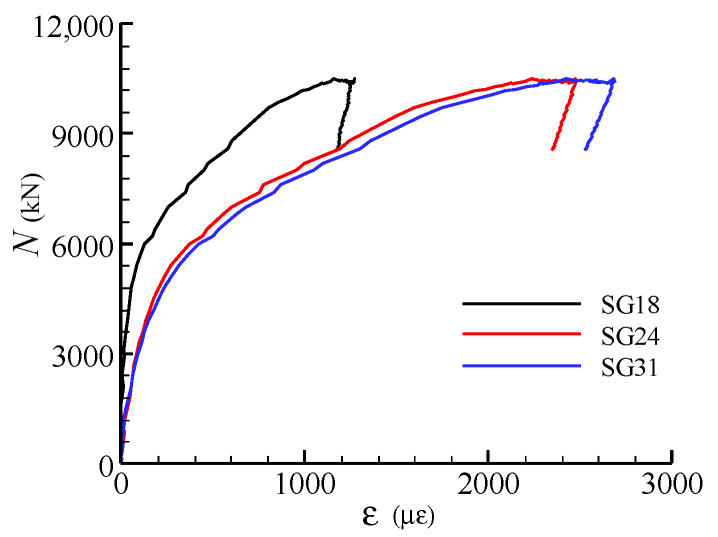
Load-strain curves for steel meshes at different layers in specimen SC1.

Figure 8 shows the load-strain curves for the steel meshes at different layers in specimen SC1. The strain gauges were arranged at the centre of the cross-section of the joints (see Figure 3). It can be seen that the strains in the steel meshes at the mid-height of the joint are larger than those in the upper and lower layers. Besides, the steel meshes reach their tensile yield strains at the peak load except the one located in the first layer (SG18).

**Figure 9:**
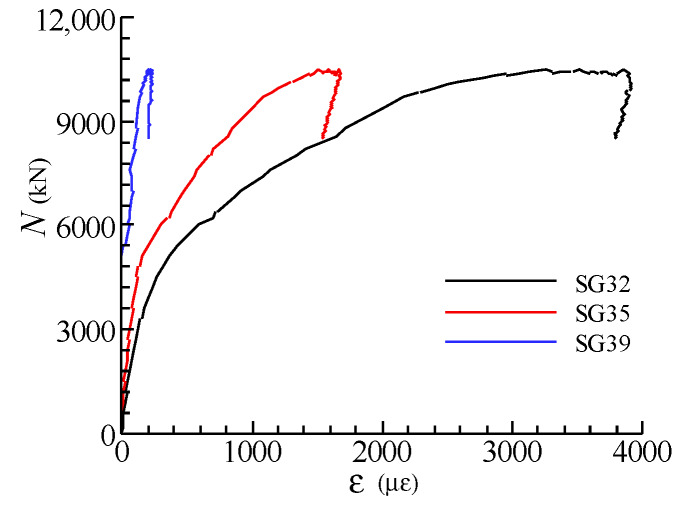
Load-strain curves for steel meshes at different plan positions in specimen SC1.

Figure 9 shows the load-strain curves for the steel mesh at the third layer with different plan positions in specimen SC1. It can be observed that the strains which are closer to the central section exhibit more serious tensile deformation. In addition, the steel meshes suffered from small compressive strain at the outermost positions (SG32) in the early loading stage.

**Figure 10:**
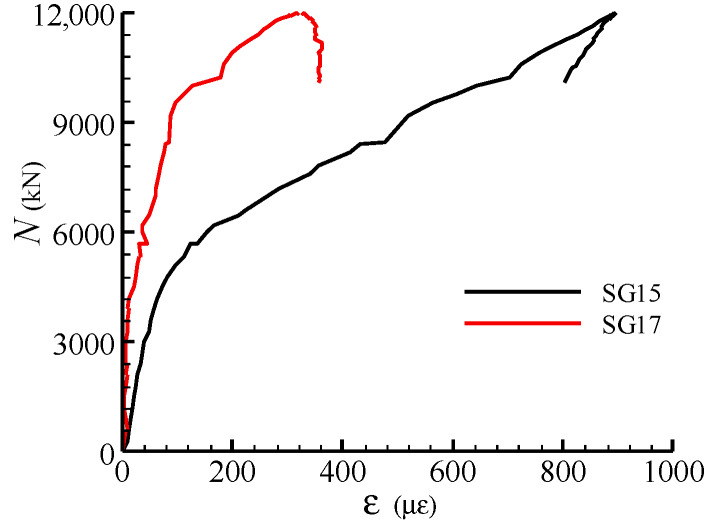
Load-strain curves for longitudinal reinforcing bars of the reinforced concrete (RC) beams.

Figure 10 illustrated the load-strain histories for the longitudinal bars in RC frame beam for specimen SC2. It can be found that the tensile strain of the longitudinal bars is 900 με at the peak loads and did not reach its tensile yield strain, which indicates that the longitudinal bars could provide favourable but limited confinement to the concrete of the joint.

### 2.4. Summary for the Series I Tests

**Figure 11:**
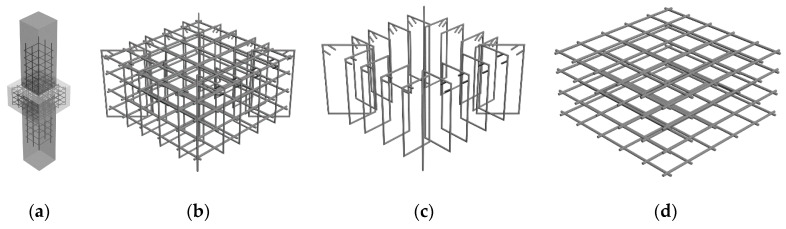
New type of joint between square concrete filled steel tube (CFST) columns and RC beams (**a**) Joints; (**b**) Steel cage of joint; (**c**) Radial stirrups; (**d**) Multi layers of steel meshes.

According to the experimental results and analysis of the Series I tests, it can be concluded that the axial load carrying capacity of the joint can be higher than those of the CFST columns by proper design, which verifies the feasibility of the innovative joint. However, the multi-layers of steel meshes in the joint is difficult to rig up because of the lack of efficient supports. Moreover, square CFST columns are extensively used as structural members due to aesthetic consideration. In order to improve the construction operability of the joints, an improved joint detailing specifically for square CFST columns is developed. In the improved joint detailing, the stirrups were added in and arranged radially around the multi-layers of steel meshes to assemble into a steel cage, as shown in Figure 11. Therefore, the axial compression behaviour of the joint with improved detailing which comprises of the multi-layers of steel meshes and radial stirrups will be further studied in the following section.

## 3. Axial Compression Tests on Innovative Joints (Series II Tests)

### 3.1. General

**Figure 12:**
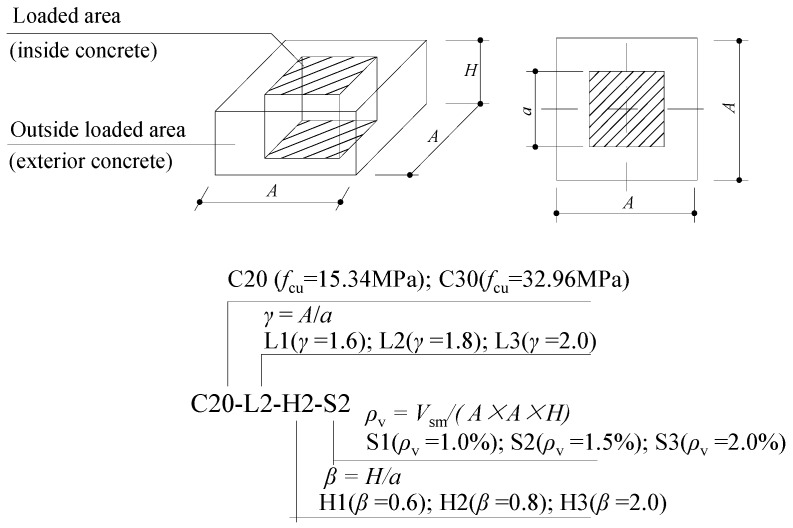
Specimens for Series II tests.

In order to study the axial compressive behaviour of the innovative joints with the improved detailing of the steel meshes, the tests of Series II were conducted subsequently. A total of 14 full-scale specimens were designed and fabricated. The details of the joint specimens are shown in Figure 12 and Table 3. The influence factors of Series II tests include the volume ratio of the steel meshes in the joint (*ρ*_v_), the dimension of the joint and the concrete strength. The test specimens were named based on the following parameters, where C20 and C30 represent the cubic compressive strengths of the concretes are 15.34 MPa and 32.96 MPa respectively, L1, L2 and L3 refer to *α* = 1.6, 1.8 and 2.0 respectively; H1, H2 and H3 indicate *β* = 0.6, 0.8 and 1.0 respectively; and S1, S2 and S3 represent *ρ*_v_ = 1.0%, 1.5% and 2.0% respectively.

The specimens were all loaded concentrically. The compression zones on surfaces of the joints were square with the side length *a* of 300 mm. In order to examine the actual bearing strengths of the joints, the upper and bottom CFST columns adopted in the Series I tests were removed and replaced by a pair of thick steel plates, with their cross-section dimensions identical with the local compression area. A total of 3 (*β* = 0.6) or 4 (*β* = 0.8 and 1.0) layers of steel meshes were horizontally and symmetrically arranged in the joint area. The diameters of the rebars in each steel mesh were determined according to the volume ratio of the steel meshes in the joint (*ρ*_v_) and the joint volume, with the detailed steel bars adopted in each layer of the steel mesh listed in Table 3. It should be noted that during the fabrication of the steel meshes, the steel bars with larger diameters were prior to being arranged in the central of the joint area. The concrete protective cover was 20 mm. The diameter of the stirrup is 6 mm. The mechanical properties of the steel bars are summarized in Table 4, while the concrete cubic strengths for C20 and C30 grades were 15.34 MPa and 32.96 MPa, respectively.

### 3.2. Experimental Test Setup

**Figure 13:**
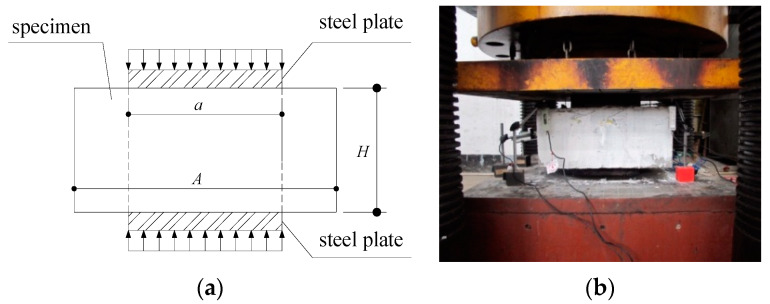
Test setup for the Series II tests (**a**) Elevation View; (**b**) Photo of the setup.

The test setup for the Series II tests is shown in Figure 13, where a pair of thick steel plates with their cross-section sizes corresponding to the local compression zone were respectively attached to the top and bottom surfaces of the specimen to simulate the actions of the CFST columns, and four LVDTs were adopted to record the displacements between the steel plates. Additionally, strain gauges were attached to the steel meshes and radial stirrups, to measure the deformations of the rebars in the joint. The loading rate utilised in the Series II tests was the same as that used for the Series II tests, as described in Section 2.2.

### 3.3. Failure Modes

**Figure 14:**
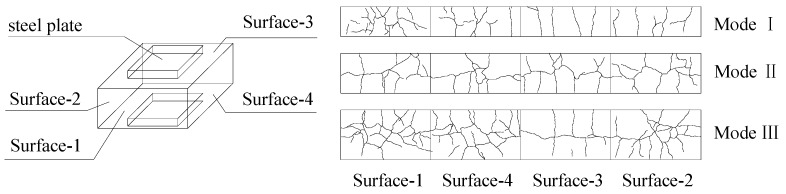
Three crack patterns on the side surfaces for the Series II specimens.

Table 5 summarises the key experimental observations, including the loads corresponding to the appearance of the first crack *N*_cr_, the loads when the maximum crack width developed to 0.2 mm *N*_0.2_ and the loads when the maximum crack width developed to 0.3 mm *N*_0.3_. It can be concluded that all the specimens had similar performance during the tests, with cracking of the concrete, yielding of the steel meshes and eventual spalling and crushing of the concrete. At the ultimate loads, several vertical penetrating cracks occurred on the surface of the joint. However, the distributions of the horizontal cracks were different with various height factor *β*. Accordingly, three different crack patterns (i.e., Mode I, Mode II and Mode III) on the surface of the specimens can be classified according to the distributions of the horizontal cracks, as shown in Figure 14. As the height factor *β* increases from 0.6 to 1.0, the horizontal cracks begin to generate, and the number of horizontal penetrating cracks gradually rises. The crack pattern modes of all the 14 specimens are summarised in Table 5.

Regarding the crack pattern of Mode I, several vertical penetrating cracks can be found on the surfaces of the joint. The horizontal cracks may appear on the surfaces of the joint, but they were not penetrating (Figure 14). The specimens with the height factor *β* of 0.6 were categorised as the crack pattern of Mode I. Taking specimen C30-L2-H1-S2 as an example to describe the experimental observation in detail, the first vertical crack appeared when the upper load reached 800 kN. The crack extended gradually and became a penetrating crack when the load reached 1200 kN. The steel meshes started to reach the yield strains as the load increased to about 4000 kN. After that, the specimen reached its maximum load-carrying capacity of 7800 kN. During the post-peak loading stage, the concretes began to spall and crush. The test was terminated when the bearing capacity of the specimen declined to 85% of its peak load. 

Regarding the crack pattern of Mode II, several vertical penetrating cracks and a main horizontal penetrating crack can be observed on each side surface of the joint (see Figure 14). The specimens with the height factor *β* of 0.8 were categorised as the crack pattern of Mode II. Taking specimen C30-L2-H2-S2 as an example to describe the experimental observation in detail, the first vertical crack occurred at the load of 1200 kN and developed into a penetrating crack at the load of 1500 kN. When the upper load reached 3300 kN, several horizontal cracks began to appear and gradually developed into a penetrating crack at the load of 5000 kN. At the load of about 6500 kN, the specimen reached its maximum load-carrying capacity. The test was ended when the upper load dropped to 85% of its peak load.

Regarding the crack pattern of Mode III, several vertical penetrating cracks and more than one horizontal penetrating cracks could be found on each side surface of the specimen (see Figure 14). The specimens with the height factor *β* of 1.0 were categorised as the crack pattern of Mode III. Taking specimen C30-L2-H3-S2 as an example to describe the experimental observation in detail, the first vertical crack appeared at the load of 900 kN. When the upper load reached 2700 kN, the first horizontal crack occurred. After that, several horizontal cracks could be observed on each side surface of the specimen and eventually generated two main horizontal penetrating cracks when the load increased to 5000 kN. The specimen reached its load-carrying capacity of 5550 kN. The test was terminated when the load dropped to 85% of its peak load.

**Figure 15:**
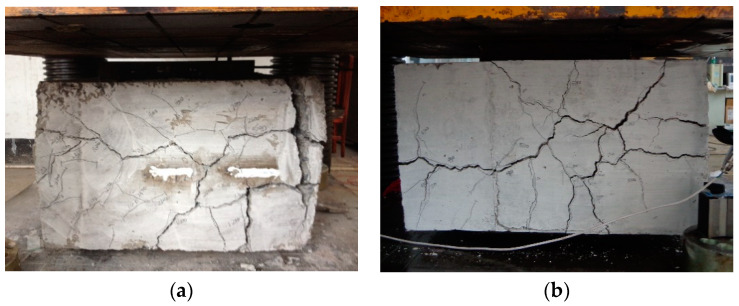
Comparison of failure modes for specimens with different concrete strengths (**a**) C20 L1 H3 S2; (**b**) C30 L2 H3 S2.

The comparison of failure modes between different concrete strengths is displayed in Figure 15. For the specimen C20-L1-H3-S2 with lower concrete strength (see Figure 15a), serious spalling and crushing of the concretes could be observed in the experiment when the specimen reached its ultimate strength, while for the specimen C30-L2-H3-S3 with higher concrete strength, the concretes of the joint remained intact at the post-peak loading stage even though serious vertical and horizontal cracks had been developed (Figure 15b).

### 3.4. Load-Deformation Curves and Load-Strain Curves

**Figure 16:**
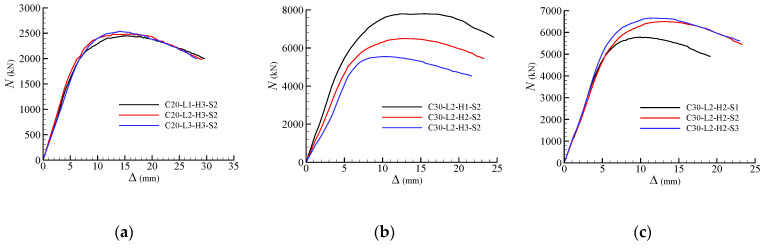
Axial load (*N*) verse displacement (Δ) relationships (**a**) Series C20 H3 S2; (**b**) Series C30 L2 S2; (**c**) Series C30 L2 H2.

The load (*N*) versus longitudinal displacement (Δ) curves for typical specimen series are plotted in Figure 16. It can be seen that the strengths of the joints were sustained or declined slowly after the peak loads, which indicates excellent ductility of the joints. Figure 16a shows the load-deformation curves for specimen series C20-H3-S2, where all the parameters are the same except for the length factor *α*. It can be observed that the curves are quite close to each other. As shown in Figure 16a and Table 5, the ultimate strengths for specimens C20-L2-H3-S2 and C20-L3-H3-S2 are 1.013 and 1.034 times higher than that of specimen C20-L1-H3-S2. It can be concluded that the increase of *α* is advantageous but limited to the peak loads of the specimens in this experiment. Figure 16b shows the load-deformation curves for specimen series C30-L2-S2. In these three specimens, the height factors *β* are 0.6, 0.8 and 1.0 for specimens C30-L2-H1-S2, C30-L2-H2-S2 and C30-L2-H3-S2, respectively. As shown in Figure 16b and Table 5, the height factor *β* is a significant parameter affecting the behaviour of the specimen. The increase of *β* could evidently reduce the initial stiffnesses and the peak loads of the specimens. Figure 16c shows the load-deformation curves for specimen series C30-L2-H2. The volume ratios of steel meshes in the joint *ρ*_v_ for specimens C30-L2-H2-S1, C30-L2-H2-S2 and C30-L2-H2-S3 are 1.0%, 1.5% and 2.0%, respectively. As shown in Figure 16c and Table 5, the peak load of the specimens C30-L2-H2-S2 and C30-L2-H2-S3 are 1.125 and 1.152 times higher than that of the specimen C30-L2-H2-S1, which states that the increase of *ρ*_v_ could significantly increase the peak strengths of the specimens. However, the strengthen efforts could be weakening once *ρ*_v_ is larger than 1.5%. In addition, the effect of *ρ*_v_ on initial stiffness of the specimen is limited.

**Figure 17:**
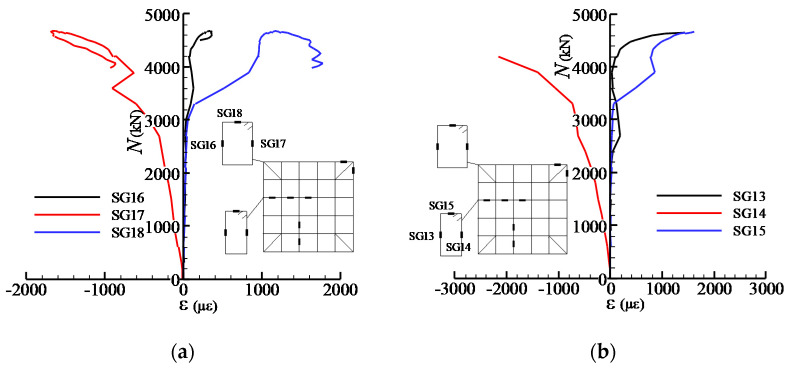
Load-strain curves for radial stirrups in specimen C30-L2-H3-S1 (**a**) SG16, SG17 and SG18; (**b**) SG13, SG15 and SG15.

The load-strain curves of the radial stirrups for a typical specimen C30-L2-H3-S1 are present in Figure 17, where the outer and upper parts of the radial stirrups were in tension, while the internal part of the stirrup was in compression. The internal part of the stirrup approximately reached compressive yield strain at the ultimate load stage, which states that the stirrup could provide certain axial compressive strength for the joint.

### 3.5. Summary for the Series II Tests

According to the experimental results and analysis of the Series II, it can be concluded that (i) the resistances of the joints were sustained or declined slowly after the peak loads, indicating excellent ductility of the joints when subjected to bearing loads, and (ii) with the decrease of height factor *β* and the increase of volume ratio of steel meshes in the joint *ρ*_v_, the axial compressive strengths of the joints significantly increased, while the increase of the length factor *α* is advantageous but limited to the peak loads of the specimens. 

## 4. Calculation of Axial Compression Capacity of the Joints

### 4.1. General

Because of the lack of existing guidelines for the design of the innovative joints, a theoretical investigation was conducted in this section to determine the axial compression resistances of the innovative joints subjected to bearing loads, based on the test observations and analysis in Section 2 and Section 3. In the following subsections, the local compression effect, the confinement effect provided by the multi-layers of steel meshes and the height effect of concrete will be first discussed. New proposal for calculating the axial compression resistances of the innovative joints subjected to bearing loads was then proposed and validated with the experimental results.

### 4.2. Effect of Local Compression

Several experimental studies have been conducted to investigate the resistances of plain concretes under local compression [17,18,19,20,21,22], and demonstrated that local compressive strengths of the plain concretes increased because the concrete outside the local compression area provided confined stress to the concretes within the local compression area, in which the degree of confinement can be quantified by using the bearing ratio (i.e., the ratio of the total surface area to the bearing area). A relationship representing the degree of confinement has been proposed by Komendant [23] and expressed as the square root function between the bearing area and the total surface area, which has been widely used in the existing international design codes, including the American Concrete Institute (ACI) design code ACI 318-08 [24] and the Chinese building codes GB 50010 [16]. In the present study, the expression specified in ACI 318-08 [24] was adopted to determine the nominal bearing strength of plain concrete, as given by Equation (1), where *N*_plain_ is the design bearing compression of unconfined concrete; *f*_c_ is the characteristic compressive cylinder strength of unconfined concrete; *A*_1_ is the loaded area; *A*_2_ is the area of the lower base of the largest frustum of a pyramid, cone or tapered wedge contained wholly within the support and having its upper base equal to the loaded area.

(1) $$N_{\mathrm{plain}}=0.85f_{c}A_{1}\sqrt{\frac{A_{2}}{A_{1}}}\;\mathrm{with}\;\sqrt{\frac{A_{2}}{A_{1}}}\leq 2.0$$

### 4.3. Confinement Effect Provided by the Multi-Layers of Steel Meshes

It is certified from the experimental results that the ductility and the bearing strength of concrete improved due to the confinement from the multi-layers of steel meshes. However, the advantage contribution of the confinement effect on the concrete strength is not included in Equation (1), as set out in ACI 318-08 [24], which would underestimate the effective compressive strength of the concrete. In order to take into account the beneficial confinement effects provided by the multi-layers of steel meshes, the confined concrete model, as proposed by Mander et al. [25], was used to quantify the degree of confinement provided by the multi-layers of steel meshes; this confined concrete model has also been used in the previous experimental and theoretical studies on the axial compressive strength of a circular through-beam joint between the CFST columns and RC beams [11], indicating that the confined concrete model proposed by Mander et al. [25] was capable of precisely predicting the compressive strength of the circular through-beam joint. The expression for calculating the confined concrete strength is given in Equation (2), where ${f^{\prime }}_{\mathrm{cc}}$ is the peak compressive strength of the confined concrete; ${f^{\prime }}_{\mathrm{co}}$ is the peak compressive strength of the unconfined concrete; ${f^{\prime }}_{\ell }$ is the effective lateral confining pressure applied by reinforcing bars.

(2) $${f^{\prime }}_{\mathrm{cc}}=\left[-1.254+2.254{\left(1+7.94\frac{{f^{\prime }}_{\ell }}{{f^{\prime }}_{\mathrm{co}}}\right)}^{0\cdot 5}-2.0\frac{{f^{\prime }}_{\ell }}{{f^{\prime }}_{\mathrm{co}}}\right]\;{f^{\prime }}_{\mathrm{co}}$$

Given that the amount of the reinforcements in both directions of the multi-layers of steel meshes are approximately the same, the effective lateral confining pressure ${f^{\prime }}_{\ell }$ is then determined by Equation (3) [25], where *f_l_* is the lateral pressure provided by the transverse reinforcements and assumed to be uniformly distributed in the concrete core; *f*_y_ is the yield strength of the reinforcing bars; *k*_e_ is the confinement effectiveness coefficient, which takes into account the reduction in the confinement effect due to the spalling off of the cover concrete. Although vertical and horizontal cracks were observed on the side surfaces of the joint specimens, the concrete in the loaded area remained intact in the experiments. Therefore, the reduction in the confinement effect due to the spalling of the cover concrete is neglected, with the confinement effectiveness coefficient *k*_e_ = 1, leading to the expression to calculate the lateral pressure given by Equation (4).

(3) $$f_{l}{}^{\prime }=k_{e}f_{l}=\frac{k_{e}\rho_{v}f_{y}}{2}$$

(4) $$f_{l}{}^{\prime }=k_{e}f_{l}=\frac{\rho_{v}f_{y}}{2}$$

### 4.4. Height Effect of Concrete

**Figure 18:**
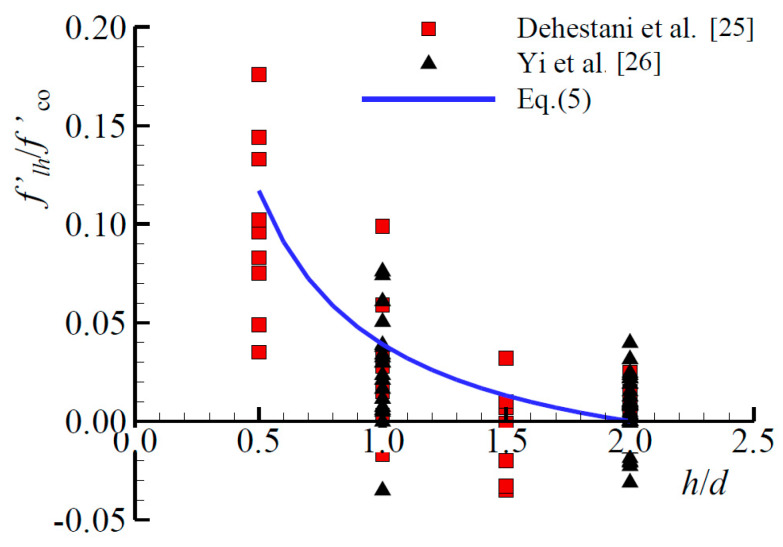
Variation in ${f^{\prime }}_{lh}/{f^{\prime }}_{\mathrm{co}}$ with *h*/*d* ratio.

Existing experimental results have demonstrated that the height factor *β* is a critical factor affecting the ultimate strength of the joint [11,20]. With the height of the specimen increasing, the ultimate strength of the specimen declined rapidly, as evident in Section 3.4. The reason for the height effect of concrete can be explained by the friction generated at the surface between the steel plates and the concretes. The friction gradually transferred from the contact surface to the mid-height cross-section of the specimen, which plays a role of confinement and thus strengthens the compressive strength of the concrete. In order to quantify the degree of confinement at the mid-height cross section of the joint due to the friction effect, a regression analysis was conducted, based on the existing experimental results on the height effect of plain concrete. First, a total of 99 experimental data on the height effect of concrete were collected from the existing experiments carried out by Dehestani et al. [26] and Yi et al. [27]. The lateral pressure ${f^{\prime }}_{\ell h}$ for the height effect of each data can then be derived by substituting the corresponding unconfined concrete strengths and the confine concrete strengths into Equation (2). The ratio of ${f^{\prime }}_{\ell h}/{f^{\prime }}_{\mathrm{co}}$ for each data was plotted against the corresponding *h*/*d* ratio and displayed in Figure 18, in which *d* is the width of the local compression zone. As shown in Figure 18, the increase of *h*/*d* ratio results in a downtrend of ${f^{\prime }}_{\ell h}/{f^{\prime }}_{\mathrm{co}}$ and the regression equation of lateral pressure for the height effect of concrete can be obtained and given by Equation (5).

(5) $$\frac{{f^{\prime }}_{\ell h}}{{f^{\prime }}_{\mathrm{co}}}=\frac{1}{12.820h/d}-0.039,\;0.5<h/d\leq 2$$

In Equation (5), it is discovered that ${f^{\prime }}_{\ell h}/{f^{\prime }}_{\mathrm{co}}$ = 0 when *h*/*d* = 2, which represents the frictional force between the loading plates and specimens is neglected if the height-to-width ratios of the concrete specimens are greater than 2. According to Equations (2) and (5), when *h*/*d* = 1, the value of ${f^{\prime }}_{\ell h}/{f^{\prime }}_{\mathrm{co}}$ is equal to 0.8, which were consistent with the conversion relationship of BSI for the concrete compressive strength between the cylindrical specimen and the cubic specimen [28].

### 4.5. Prediction and Discussion

Through considering the local compression effect, the confinement effect provided by the multi-layers of steel meshes and the height effect of concrete, the formulas for calculating the compressive resistances of the joints are summarised in Equations (6)–(8).

(6) $$N_{\mathrm{cal}}=0.85{f^{\prime }}_{\mathrm{cc}}A_{1}\sqrt{\frac{A_{2}}{A_{1}}}$$

(7) $${f^{\prime }}_{\mathrm{cc}}=\left[-1.254+2.254{\left(1+7.94\frac{{f^{\prime }}_{\ell }}{{f^{\prime }}_{\mathrm{co}}}\right)}^{0\cdot 5}-2.0\frac{{f^{\prime }}_{\ell }}{{f^{\prime }}_{\mathrm{co}}}\right]\;{f^{\prime }}_{\mathrm{co}}$$

(8) $$f_{l}{}^{\prime }=\frac{\rho_{v}f_{y}}{2}+(\frac{1}{12.820H/a}-0.039){f^{\prime }}_{\mathrm{co}}$$

Assessment of the accuracy of the new proposal was then conducted, through the comparisons between the experimental ultimate strengths *N*_u_ and the predicted results *N*_cal_, as listed in Table 1 and Table 5. It can be found that the resistance predictions *N*_cal_ for the joint specimens in Series I are higher than the corresponding experimental counterparts, which could be attributed to the fact that all the specimens in Series I failed by the CFST columns, and the ultimate strengths of the joints were higher than those of the CFST columns. For Series II, the resistance predictions derived by Equations (6)–(8) are in good agreement with the experimental ones for normal strength concrete (C30), with a mean value and standard deviation of *N*_cal_/*N*_u_ equal to 1.038 and 0.100, respectively. However, the ultimate strengths were overestimated for the specimen series with low strength concrete (C20). It may be because the outer concrete of the specimens with lower concrete strength was prematurely spalled and crushed when the specimens were reaching their ultimate strengths, which would weaken the favourable effect of local compression.

Therefore, the calculation model for the ultimate strengths of the joints subjected to bearing loads for practical engineering design is recommended herein. The confinement effect provided by the multi-layers of steel meshes and the height effect of concrete are considered in the model, while the effect of local compression is neglected because of its uncertainty, leading to the final formulations for the design of the ultimate strengths of the square joints given by Equations (9) and (10).

(9) $$N_{\mathrm{design}}=\left[-1.254+2.254{\left(1+7.94\frac{{f^{\prime }}_{\ell }}{{f^{\prime }}_{\mathrm{co}}}\right)}^{0\cdot 5}-2.0\frac{{f^{\prime }}_{\ell }}{{f^{\prime }}_{\mathrm{co}}}\right]\;{f^{\prime }}_{\mathrm{co}}A_{1}$$

(10) $$f_{l}{}^{\prime }=\frac{\rho_{v}f_{y}}{2}+(\frac{1}{12.820H/a}-0.039){f^{\prime }}_{\mathrm{co}}$$

**Figure 19:**
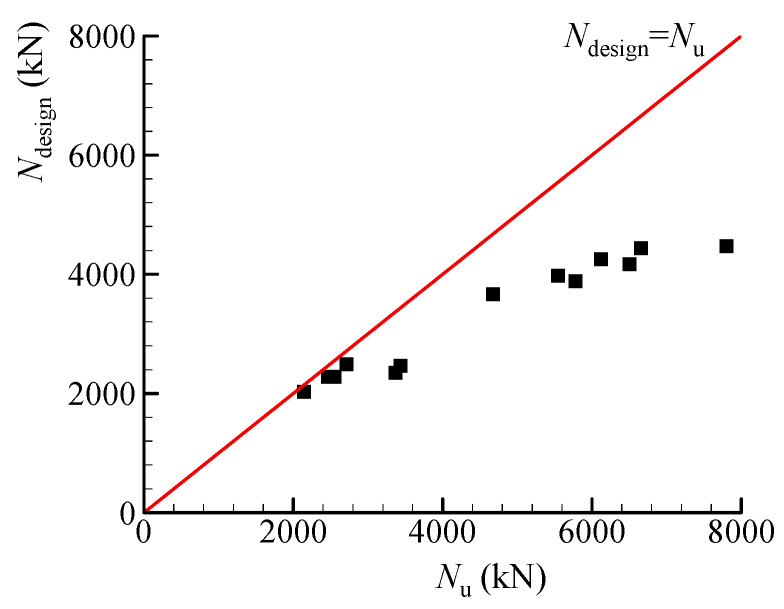
Comparison of the calculated load for design (*N*_design_) and experimental load (*N*_u_).

Figure 19 shows the comparison between the calculated results obtained by Equations (9) and (10) and experimental results of Series II tests, where the calculated strengths are relatively accurate and conservative in contrast to the experimental strengths. 

## 5. Conclusions

The axial compressive behaviour of square through-beam joint between CFST column and RC beams with multi-layers of steel meshes was experimentally and theoretically investigated in this paper. The following conclusions can be drawn based on the results of this study.

The first stage of the tests included three full-scale innovative joint specimens, indicating that the innovative joint is capable of achieving the design criterion of the ‘strong joint-weak member’ with appropriate designs. 

The second stage of the tests involved 14 innovative joint specimens with the improved detailing to study the effects of the geometric size of the joint, concrete strength and volume ratio of the steel meshes on the bearing strengths of the joints. It is shown that by increasing the height factor *β* of the joint specimens from 0.6 to 1.0, the axial compression strengths of the joints significantly decreased with a maximum reduction ratio in strength equal to 28.9%. The increase of the volume ratio of the steel meshes in the joint *ρ*_v_ is advantageous for the axial compression strengths of the joints. As *ρ*_v_ increased from 1.0 to 2.0, the axial compression strengths of the joints increased by 26.4% and 15.2% for the specimens with concrete grades of C20 and C30, respectively. The increase of the length factor *α* is advantageous but limited to the peak loads of the specimens. 

Because of the lack of existing design methods for the innovative joints, new design expressions were proposed to calculate the axial compression resistances of the innovative joints subjected to bearing loads, with the local compression effect, the confinement effect provided by the multi-layers of steel meshes and the height effect of the concrete considered. The strength predictions of the joints are in good agreement with the experimental ones for normal strength concrete. Through the discussion of the calculated results, recommended formulations for the design of the joints were proposed, which is capable of providing accurate and safe resistance predictions for the innovative joints. However, because of the limited range of variables chosen for the specimens in the tests, extensive applications of the proposed calculation method should be verified by further experimental researches.

## Figures and Tables

**Table 1:** Specimen parameters and the experimental and calculated loads for Series I tests.

Specimen	*D* × *t* (mm × mm)	*A* × *A* × *H* (mm × mm × mm)	*f*_cu,u_ (MPa)	*f*_cu_ (MPa)	*f*_cu,l_ (MPa)	*d*_bar_ (mm)	*ρ*_v_ (%)	*N*_cr_ (kN)	*N*_u_ (kN)	*N*_cr_ */N*_u_	*N*_cal_ (kN)	*N*_cal_ */N*_u_
SC1	500 × 6	1000 × 1000 × 600	46.3	40.8	44.7	11	1.65	6000	10,534	0.57	20,112	1.91
SC2	500 × 6	1000 × 1000 × 600	46.3	40.8	44.7	8.5	0.99	5300	12,004	0.44	18,395	1.53
SC3	500 × 6	1000 × 1000 × 600	46.3	40.8	44.7	5.5	0.41	5100	11,404	0.45	15,954	1.40

Notes: *D* and *t* are diameter and thickness of steel tube; *A* and *H* are width and height of the joint, respectively; *f*_cu,u_, *f*_cu_ and *f*_cu,l_ are the cubic compressive strengths of the concrete for upper column, joint area and lower column, respectively; *d*_bar_ is the diameter of rebar in the steel meshes; *ρ*_v_ is the volume ratio of steel mesh in the joint, which is calculated as *V*_sm_/(*A* × *A* × *H*); *V*_sm_ is the total volume of steel meshes in the joint; *N*_cr_ is the load when the first crack appeared; *N*_u_ is the peak load of the specimen; *N*_cal_ is the calculated strength obtained by Equations (6)–(8).

**Table 2:** Measured material properties of reinforcing bars and steel tube for Series I test.

Member	Bar Φ5.5	Bar Φ8	Bar Φ8.5	Bar Φ11	Bar Φ22	Bar Φ25	Steel Tube
Yield strength *f*_y_ (MPa)	517	318	510	448	386	368	330
Ultimate strength *f*_u_ (MPa)	578	460	608	540	575	575	460

**Table 3:** Specimen parameters for Series II tests.

Specimen	*A* × *A* × *H*	*a* × *a*	*f*_cu_	*γ*	*β*	*ρ_v_*	Layers of Steel Meshes	1st Layer Steel Mesh	2nd Layer Steel Mesh	3rd Layer Steel Mesh	4st Layer Steel Mesh
	(mm × mm × mm)	(mm)	(MPa)			(%)					
C20-L1-H3-S2	480 × 480 × 300	300 × 300	15.35	1.6	1.0	1.5	4	6Φ8	6Φ8	6Φ8	6Φ8
C20-L2-H1-S2	540 × 540 × 180	300 × 300	15.35	1.8	0.6	1.5	3	6Φ8	4Φ6 + 2Φ8	6Φ8	-
C20-L2-H2-S2	540 × 540 × 240	300 × 300	15.35	1.8	0.8	1.5	4	6Φ8	3Φ6 + 3Φ8	3Φ6 + 3Φ8	6Φ8
C20-L2-H3-S1	540 × 540 × 300	300 × 300	15.35	1.8	1.0	1.0	4	6Φ6	5Φ8 + 1Φ6	5Φ8 + 1Φ6	6Φ6
C20-L2-H3-S2	540 × 540 × 300	300 × 300	15.35	1.8	1.0	1.5	4	6Φ6	3Φ8 + 3Φ10	3Φ8 + 3Φ10	6Φ6
C20-L2-H3-S3	540 × 540 × 300	300 × 300	15.35	1.8	1.0	2.0	4	6Φ10	1Φ8 + 5Φ10	1Φ8 + 5Φ10	6Φ10
C20-L3-H3-S2	600 × 600 × 300	300 × 300	15.35	2.0	1.0	1.5	4	6Φ8	5Φ10 + 1Φ8	5Φ10 + 1Φ8	6Φ8
C30-L2-H1-S2	540 × 540 × 180	300 × 300	32.96	1.8	0.6	1.5	3	6Φ8	4Φ6 + 2Φ8	6Φ8	-
C30-L2-H2-S1	540 × 540 × 240	300 × 300	32.96	1.8	0.8	1.0	4	6Φ6	5Φ6 + 1Φ8	5Φ6 + 1Φ8	6Φ6
C30-L2-H2-S2	540 × 540 × 240	300 × 300	32.96	1.8	0.8	1.5	4	6Φ8	3Φ6 + 3Φ8	3Φ6 + 3Φ8	6Φ8
C30-L2-H2-S3	540 × 540 × 240	300 × 300	32.96	1.8	0.8	2.0	4	6Φ10	4Φ8 + 2Φ6	4Φ8 + 2Φ6	6Φ10
C30-L2-H3-S1	540 × 540 × 300	300 × 300	32.96	1.8	1.0	1.0	4	6Φ6	5Φ8 + 1Φ6	5Φ8 + 1Φ6	6Φ6
C30-L2-H3-S2	540 × 540 × 300	300 × 300	32.96	1.8	1.0	1.5	4	6Φ8	3Φ8 + 3Φ10	3Φ8 + 3Φ10	6Φ8
C30-L2-H3-S3	540 × 540 × 300	300 × 300	32.96	1.8	1.0	2.0	4	6Φ10	1Φ8 + 5Φ10	1Φ8 + 5Φ10	6Φ10

Notes: *A* and *H* are width and height of the joint, respectively; *f*_cu_ is the cubic compressive strength of the concrete; *γ* is the length factor, which is calculated as *A*/*a*; *β* is the height factor and is calculated as *β* = *H*/*a*, *ρ*_v_ is the volume ratio of steel mesh in the joint and is calculated as *V*_sm_/(*A* × *A* × *H*), where *V*_sm_ is the total volume of steel meshes in the joint.

**Table 4:** Measured material properties of reinforcing bars for Series II tests.

Member	Series C20			Series C30		
	BarΦ6	Bar Φ8	Bar Φ10	Bar Φ6	Bar Φ8	Bar Φ10
Yielding strength *f*_y_ (MPa)	259.9	472	422	260	300	340
Ultimate strength *f*_u_ (MPa)	430	536.1	480.4	440	340	430

**Table 5:** Ultimate strength, characteristic load and failure modes for Series II tests.

Specimens	*N*_cr_ (kN)	*N*_cr_/*N*_u_	*N*_0.2_ (kN)	*N*_0.2_/*N*_u_	*N*_0.3_ (kN)	*N*_0.3_/*N*_u_	*N*_u_ (kN)	Crack Mode	*N*_cal_ (kN)	*N*_cal_/*N*_u_
C20-L1-H3-S2	300	0.122	800	0.325	1000	0.406	2465	III	3097	1.26
C20-L2-H1-S2	400	0.116	1100	0.320	1500	0.437	3435	I	3763	1.10
C20-L2-H2-S2	600	0.178	1400	0.416	1500	0.445	3369	II	3592	1.07
C20-L2-H3-S1	600	0.280	1200	0.559	1400	0.652	2146	III	3103	1.45
C20-L2-H3-S2	600	0.240	1000	0.400	1400	0.560	2498	III	3484	1.39
C20-L2-H3-S3	600	0.221	750	0.277	1000	0.369	2712	III	3813	1.41
C20-L3-H3-S2	600	0.235	1050	0.412	1400	0.549	2550	III	3871	1.52
C30-L2-H1-S2	800	0.103	1100	0.141	1800	0.231	7801	I	6845	0.88
C30-L2-H2-S1	900	0.156	2100	0.363	2700	0.467	5782	II	5938	1.03
C30-L2-H2-S2	1200	0.184	2400	0.369	3300	0.507	6505	II	6380	0.98
C30-L2-H2-S3	1200	0.180	2400	0.360	2700	0.405	6659	II	6789	1.02
C30-L2-H3-S1	900	0.193	1500	0.321	2400	0.514	4673	III	5612	1.20
C30-L2-H3-S2	900	0.162	1500	0.270	1800	0.324	5550	III	6081	1.10
C30-L2-H3-S3	900	0.147	1500	0.245	1800	0.294	6122	III	6512	1.06

Notes: *N*_cr_ is the load when the first crack appeared, *N*_u_ is the peak load of specimen, *N*_0.2_ and *N*_0.3_ are the loads when the maximum crack width developed to 0.2 mm and 0.3 mm respectively, *N*_cal_ is the calculated strength obtained by Equations (6)–(8).
